# Lithium Chloride Dependent Glycogen Synthase Kinase 3 Inactivation Links Oxidative DNA Damage, Hypertrophy and Senescence in Human Articular Chondrocytes and Reproduces Chondrocyte Phenotype of Obese Osteoarthritis Patients

**DOI:** 10.1371/journal.pone.0143865

**Published:** 2015-11-30

**Authors:** Serena Guidotti, Manuela Minguzzi, Daniela Platano, Luca Cattini, Giovanni Trisolino, Erminia Mariani, Rosa Maria Borzì

**Affiliations:** 1 Laboratorio di Immunoreumatologia e Rigenerazione Tessutale, Istituto Ortopedico Rizzoli, Bologna, Italy; 2 Dipartimento di Scienze Mediche e Chirurgiche-DIMEC, Università di Bologna, Bologna, Italy; 3 Dipartimento di Scienze Biomediche e Neuromotorie-DIBINEM, Università di Bologna, Bologna, Italy; 4 Dipartimento RIT, Laboratorio RAMSES, Istituto Ortopedico Rizzoli, Bologna, Italy; 5 Chirurgia ricostruttiva articolare dell’anca e del ginocchio, Istituto Ortopedico Rizzoli, Bologna, Italy; University of Newcastle, UNITED KINGDOM

## Abstract

**Introduction:**

Recent evidence suggests that GSK3 activity is chondroprotective in osteoarthritis (OA), but at the same time, its inactivation has been proposed as an anti-inflammatory therapeutic option. Here we evaluated the extent of GSK3β inactivation *in vivo* in OA knee cartilage and the molecular events downstream GSK3β inactivation *in vitro* to assess their contribution to cell senescence and hypertrophy.

**Methods:**

*In vivo* level of phosphorylated GSK3β was analyzed in cartilage and oxidative damage was assessed by 8-oxo-deoxyguanosine staining. The *in vitro* effects of GSK3β inactivation (using either LiCl or SB216763) were evaluated on proliferating primary human chondrocytes by combined confocal microscopy analysis of Mitotracker staining and reactive oxygen species (ROS) production (2',7'-dichlorofluorescin diacetate staining). Downstream effects on DNA damage and senescence were investigated by western blot (γH2AX, GADD45β and p21), flow cytometric analysis of cell cycle and light scattering properties, quantitative assessment of senescence associated β galactosidase activity, and PAS staining.

**Results:**

*In vivo* chondrocytes from obese OA patients showed higher levels of phosphorylated GSK3β, oxidative damage and expression of GADD45β and p21, in comparison with chondrocytes of nonobese OA patients. LiCl mediated GSK3β inactivation *in vitro* resulted in increased mitochondrial ROS production, responsible for reduced cell proliferation, S phase transient arrest, and increase in cell senescence, size and granularity. Collectively, western blot data supported the occurrence of a DNA damage response leading to cellular senescence with increase in γH2AX, GADD45β and p21. Moreover, LiCl boosted 8-oxo-dG staining, expression of IKKα and MMP-10.

**Conclusions:**

In articular chondrocytes, GSK3β activity is required for the maintenance of proliferative potential and phenotype. Conversely, GSK3β inactivation, although preserving chondrocyte survival, results in functional impairment via induction of hypertrophy and senescence. Indeed, GSK3β inactivation is responsible for ROS production, triggering oxidative stress and DNA damage response.

## Introduction

Healthy articular adult chondrocytes live in a maturation arrested state keeping a tight and low turnover of extracellular matrix proteins. Osteoarthritis (OA) is the result of the loss of this maturational arrested state [1] under the effects of a number of different pathogenetic mechanisms.

GSK3, an enzyme with many functions in intracellular signaling and metabolic control of the cell [2] is among the molecular constraints which keep chondrocytes in the “arrested state”. GSK3 belongs to the β-catenin degradation complex and acts by keeping an inactive phosphorylated form of β-catenin thus preventing its nuclear translocation and transcriptional activation of TCF/LEF complex. A tightly regulated level of β-catenin signaling must be guaranteed for an healthy articular cartilage [3]. A fine tuning of GSK3 activity is required for chondrogenesis and skeletal development. Despite functional redundancy for GSK3α and β in murine chondrocyte differentiation [4], the different phenotypes of global GSK3-α or β knockout indicated a more pivotal role for GSK3β that is also selectively expressed in articular chondrocytes [5].

Inhibition of GSK3 activity achieved by phosphorylation of serine-21 or serine-9 in isoform α and β, respectively, is a key event in chondrocyte maturation within “temporary cartilage” in skeletal development, under the control of regulatory kinases which drive the process towards hypertrophy and terminal differentiation. An investigation of GSK3β activity in human OA tissues could help in understanding the relevance of this pathway in the homeostasis of “permanent cartilage” and particularly in correlation with metabolic risk factors. Previous studies have pinpointed that human OA tissues over-express Smurf2 [6] whose conditional over-expression in mice is followed by inhibition and proteasomal degradation of GSK3β, upregulation of β-catenin and articular cartilage degeneration [7].

Metabolic Syndrome (MetS: overweight, hypertension, dyslipidaemia and impaired glucose tolerance) is a global epidemic, affecting 23% of the general population with more than 2.5 fold prevalence in OA patients [8]. MetS indeed greatly worsen the risk of occurrence and progression of knee OA [9] and, recently, BMI has been pointed at as a significant predictor of knee OA [10]. MetOA is now recognized as having a peculiar pathogenesis compared to other OA phenotypes [11].

In the present study, we investigated the extent of GSK3β inactivation in OA knee cartilage explants. We found occurrence of articular chondrocytes with inactive GSK3β in obese patients thus hinting at GSK3β as one potential mechanism whereby metabolic factors impact on OA. The effects of GSK3β inactivation were investigated in vitro using primary human chondrocytes. GSK3β inactivation (LiCl, SB216763, gene silencing strategies, insulin) consistently showed dramatic effects on proliferation. With regards to the underlying molecular mechanisms, LiCl mediated GSK3β inactivation increased mitochondrial ROS production that led to oxidative damage (increased 8-oxo-deoxyguanosine), DNA damage response (increased expression of γH2AX and growth arrest and DNA damage–inducible protein 45β **(**GADD45β**))** and cell senescence (transient S phase arrest, increased expression of the senescence marker p21, SA-β galactosidase and PAS staining).

These findings provide a link between metabolic factors and osteoarthritis, via GSK3β inactivation which promotes at the same time survival, hypertrophy and senescence of articular chondrocytes and question the use of LiCl as a drug for OA treatment.

## Materials and Methods

Preclinical research involving human OA patient knee cartilage samples at the Rizzoli Orthopaedic Institute was carried out in compliance with the Helsinki declaration, and subjected to the approval of the ethics committee/institutional review board of the Institute (‘‘Comitato Etico dell’Istituto Ortopedico Rizzoli”), which included documentation of written patient consent forms. After the retrieval of arthroplasty-derived tissues, all patient identifiers were removed and the samples were coded by arbitrary designations to distinguish them solely for experimental purposes.

### Cartilage explants

Osteochondral specimen were established from seven patients with detailed characterization of metabolic features [8]. For some patients multiple samples were utilized. 5 out of these 7 patients were obese [body mass index (BMI) over 30]. After removal of most of the subchondral bone, the cartilage cylinders were embedded in OCT, snap frozen and kept at-80°C until processing with immunohistochemistry or immunofluorescence essentially as described in [12]. These cartilage samples were graded 1–2 according to Safranin O staining [13] and with viable cells.

Control experiments were also carried out on four samples derived from disease-free cartilage of patients who had underwent leg amputation for bone tumors.

A first screening was carried out on normal and osteoarthritic cartilage to detect expression and subcellular localization of: phospho-GSK3β [clone EPR2286Y, Millipore]. Then, since normal cartilage was phospho-GSK3β negative, only OA samples were analysed to detectexpression of 8-hydroxy-2’-deoxyguanosine (8-oxo-dG, clone 2E2, Trevigen), GADD45β [sc-8776, Santa Cruz Biotechnology (SCBT)], p21 (sc-756, SCBT), senescence associated β-galactosidase (sc-19119, SCBT), in the articular cartilage tissue from superficial to deep zones.

The subcellular localization of phospho-GSK3β or GADD45β was evaluated by reference to nuclear counterstaining (Sybr green 1:10,000, Molecular Probes).

To provide a quantitative assessment of the staining pattern, an image analysis was carried out. Two 20x fields for each layer (superficial, intermediate and deep) of the cartilage sections under analysis were examined for each patient. The number of cells was automatically counted by means of the DAPI nuclear counterstaining (selected to avoid interference with the evaluation of the intensity of the Fast Red substrate, that could arise in the case of colorimetric nuclear counterstaining) and then the cells underwent an automatic analysis procedure exploiting the NIS software (NIKON). A threshold was set in order to take into account only cells above a given staining intensity and to establish the cell percentage above that threshold. These percentages are then reported separately for obese and non obese patients showing the different staining levels for superficial, mid and deep cartilage layers.

### Chondrocyte cultures

Primary chondrocytes were obtained from 14 OA patients [12], expanded in culture up to passage 1 (p1) and then used as described below. We chose OA chondrocytes to study the effects of GSK3β inactivation *in vitro* since LiCl has been recently proposed as a therapeutic option for the treatment of this disease [14].

#### Time-lapse evaluation of ROS production downstream GSK3β inactivation

Chondrocytes were plated in Petri dishes with 0.17 mm thin glass well (Cell Culture Dish, World Precision Instruments Germany GmbH), cultured for 72 hours, and then either left unstimulated or treated for 4 hours with 5 or 10 mM LiCl or with 10μM SB216763, that behaves as a rather selective inhibitor for GSK3 at that concentration [15]. Mitochondrial involvement was analysed by “time lapse” 30 μM 2',7'-Dichlorofluorescein diacetate (DCHF-DA) ROS staining overlapping with Mitotracker Orange CMTMRos (a mitochondrial staining, Molecular Probes) along with Hoechst 33258 nuclear counterstaining. Noteworthy, DCHF-DA is still considered one of the most versatile indicators of “generalized cellular oxidative stress” [Molecular Probes’s A guide to fluorescent probes and labeling technologies” 11ty edition (2010)], yielding quantitative imaging results essentially similar to other more recently developed probes [16]. The acquisition of signals of cells in different conditions was taken at comparable stimulus and probe incubation times and with the same instrument settings for excitation and acquisition.

Fluorescent signals were acquired by confocal microscopy as in [12].

#### Effects of GSK3β inhibition on cell growth

Chondrocyte cultures from 14 different patients were used to investigate the effect of GSK3β inhibition on cell growth, cell cycle and senescence. Cells were plated at low density (10,000–15,000 cells per cm2) to rule out biased evaluation of senescence [17].

After 72 hours, cultures were kept either unstimulated or stimulated for 8, 16 and 24 hours with GSK3β inhibitors: 5 mM LiCl or 10μMSB216763 [15] or 33 nM insulin [18]. At the end, cells were recovered for western blot analysis or fixed (10 min at RT with 2% PFA) for Flow Cytometry or detection of senescence associated β-galactosidase (SA-βGal) activity and PAS staining. The cell number of the samples stimulated with GSK3β inhibitors was normalized to that of the control to obtain both the “normalized count” (each count referred to that of the control at 8 hours according to the formula: count/countNS8h) as well as the “percentage of decreased count” (each count referred to that of the control at each time, according to the formula: (countGSK3βinhibitor-countNS)/countNS*100).

#### Effects of GSK3β inhibition on cell cycle, scatter properties and 8-oxo-dG

Flow cytometry was employed to evaluate cell cycle by mean of DNA staining (Sytox green, Molecular Probes, at 5μM) of cells previously fixed with 2% PFA, post-fixed with 10μl methanol and RNAse treated (2.5 U RNAse One, Promega plus 100μg/ml RNAse A, Sigma). Analyses were performed using a FACS Canto II flow cytometer (BD).

Light scattering properties of the cells were analyzed by assessing both the forward scatter (FSC) as a mean to evaluate the cell size and therefore the hypertrophy promoting activity of LiCl as well as the side scatter (SSC), which correlates with granularity which increases in cell senescence. The median values of several thousands of cells were obtained and separated for each cell cycle phase for both control and LiCl treated cells, and normalized to the median size of control cells in the G1 phase. The extent of oxidative damage was also assessed by staining accumulation of 8-oxo-dG adducts. Samples were compared taking into account the Mean Channel of Fluorescence Intensity (MCFI) increment, i.e. the difference between the median channel of fluorescence intensity of the cells stained for 8-oxo-dG and that of the same cells probed with the negative control (isotype IgG2 control).

#### Effects of GSK3β inhibition on cell senescence and hypertrophy

Senescence was tested conventionally by the assessment of SA-βGal activity [17] (Senescent Cells Staining kit, Sigma) while progressed differentiation and hypertrophy were tested by scoring an increased glycogen content (PAS staining, SIGMA) [19]. Nearly 10,000 cells were cytospun on a glass slide and processed as recommended by the manufacturer. Then, cells were treated with SyBr Green nuclear counterstaining to undergo an automatic analysis procedure exploiting the NIS software. A threshold was set in order to take into account only cells above a given staining intensity and to establish the cell percentage above that threshold. At least four fields (with 40–160 cells each) were counted for each condition.

#### Real-time polymerase chain reaction

Following treatments, cells from three patients were also dedicated for REAL TIME PCR analysis. RNA was extracted with Trizol (Invitrogen) and processed essentially as described in [20] with values normalized to GAPDH mRNA expression according to the 2^-ΔCt^ method [21].

Primers were as follows: GAPDH (GenBank:NM_002046, forward 579–598 and reverse 701–683); IKKα/CHUK (GenBank:NM_001278, forward 1803–1826 and reverse 1865–1888); MMP10 (GenBank:NM_002425.2, forward 1278–1298 and reverse 1472–1449).

#### Immunoblotting

Proteins induced upon DNA damage were evaluated by western blotting, loading protein lysates corresponding to 150,000 cells, essentially as described in [22]. Signals were revealed with ECL Select kit (GE Healthcare), using the CCD camera acquisition system of Image Station 4000 MM and Carestream Molecular Imaging Software 5.0. (Carestream Health, Inc.). Semi-quantitative analysis of bands was performed considering “optical density” values and using QuantityOne software (BioRad). Fold changes were calculated for each time point on control samples put as 1. In search for a correlation between different proteins, fold change-values for all specimens were pulled.

Experiments were designed to kinetically assess the correlated protein expression of phospho-GSK3β (D85E12, Cell Signaling Technology), total GSK3β (clone 27C10, Cell Signaling Technology), γH2AX (07–164, Upstate–Millipore), GADD45β (sc-8776, SCBT), cyclin-dependent kinase inhibitor p21 (sc-756, SCBT) and caspase 3 (sc-7148, SCBT). IKKα was detected by clone B78-1, BD Pharmingen. Monoclonal anti-GAPDH (clone 6C5, Chemicon–Millipore) or beta-actin (clone AC-74, Sigma) served as loading controls. Experiments were carried out with cells from at least 6 different patients.

#### Small interfering RNA-mediated gene silencing

The effects of GSK3β inhibition were also investigated with gene silencing experiments. To this end, primary human chondrocytes in a 12 well plate were transfected with 25 n*M* total of either ON-TARGET*plus* GSK3β SMARTpool reagents (Thermo Scientific Dharmacon,) or ON-TARGETplus Non-targeting Pool, in combination with Lipofectamine® RNAiMAX Transfection Reagent (Invitrogen). After transfection, the cells were left in culture for 48 hours in order to express silencing. Then, distinct wells for GSK3βsiRNA (siGSK3β) or control siRNA (siCTL) were left either untreated or treated with 5 mM LiCl or 10 μM SB216763 for 8, 16 and 24 hours. Each condition was run in duplicate. Silencing was assessed by comparing GSK3β expression at gene [by real time PCR, using a primer pair suitable to detect expression of both GSK3β transcripts (transcript 1: GenBank: NM_002093.3 and transcript 2: GenBank: NM_001146156.1): forward 1699–1719 and reverse 1828–1808]; or protein level (by Flow cytometric analysis of GSK3β expression calculated as described above, using clone 27C10, Cell Signaling).

#### Statistics

All data are presented as mean ± standard error of the mean (SEM), analyzed and graphed using GraphPad Prism version 5.00 for Windows (GraphPad Software, San Diego California USA, www.graphpad.com)

Correlation was tested by Spearman r. Comparison of groups for a categorical variable was assessed by mean of contingency table and Fisher’s exact test.

Means of groups were compared with paired Student’s t test and considered significant when P < 0.05, with *P < 0.05; **P < 0.01;***P < 0.001. Two tailed Student’s t test was used throughout.

## Results

### Inactivation of GSK3β in cartilage from OA patients

Our first aim was to carry out a preliminary investigation of the extent of GSK3β inactivation in OA cartilage samples and to point at any clinical parameters that could affect this marker.

**Fig 1:**
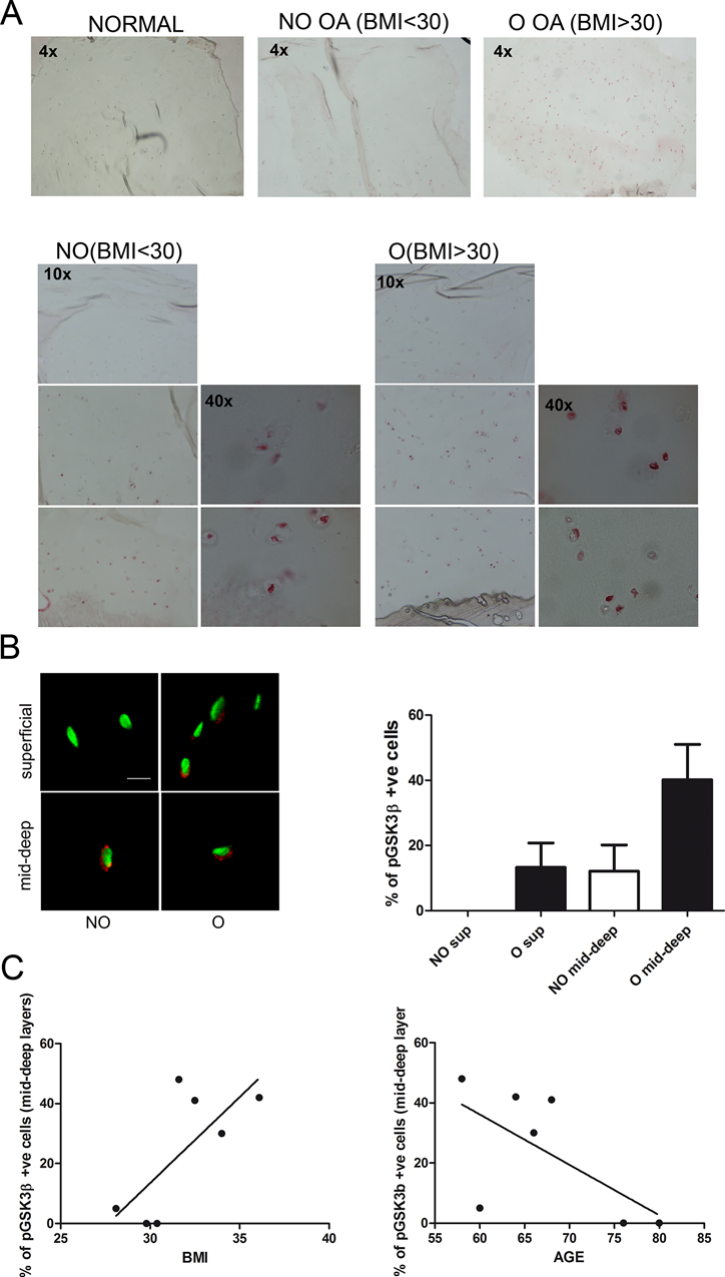
*In vivo* detection of inactive GSK3β in osteoarthritic articular chondrocytes. **1A**, Upper row (4x, original magnification): pGSK3β immunohistochemical staining in normal cartilage and in cartilage from representative examples of a non-obese and an obese patient. Lower images (10x and 40x details): pGSK3β staining in superficial, mid-deep and calcified cartilage zones, in representative samples derived from a non-obese (left) or from an obese patient (right). **1B**, High magnification images of pGSK3β staining obtained with confocal microscopy of chondrocytes in the superficial (upper row) or mid-deep layers (lower row) of cartilage derived from a non-obese (left column) or from an obese patient (right column). Bar = 10 μm. Graph: percentage of phospho-GSK3β positive cells in superficial or mid-deep layers in non obese (NO, white column) or obese patients (O, black columns) as assessed by confocal microscopy. **1C**, Left graph: percentage of phospho-GSK3β positive chondrocytes in mid-deep layers of knee cartilage, represented as a function of the Body Mass Index of the patients. Right graph: percentage of phospho-GSK3β positive chondrocytes in mid-deep layers of knee cartilage, represented as a function of the age of the patients.

The extent of phospho-GSK3β was investigated by confocal microscopy and immunohistochemistry onto a set of 15 different knee OA cartilage samples (from 7 patients; in case of replicate samples for the same patients the mean values were considered) and on samples from 4 normal subjects. Normal cartilage was negative to phospho-GSK3β staining among all layers, while OA cartilage had most phospho-GSK3β positive cells localized in mid-deep layers with an high (>30%) prevalence of positive cells in obese patients (BMI over 30) (Fig 1A and 1B and S1 File). The analysis indicated that the percentage of phospho-GSK3β positive chondrocytes showed a trend towards a positive correlation with BMI (7 patients: Spearman r = 0.631, p = 0.069, Fig 1C, left graph) and negative with the age of the patients (Spearman r = - 0.739, p = 0.066 Fig 1C, right graph). The data were also analyzed exploiting a contingency table that distinguished “obese” (BMI >30) and “non obese” patients and “phospho-GSK3β positive” (>30%) and “phospho-GSK3β negative” patients. The Fisher’s exact test was statistically significant (two tailed p value = 0.0476). phospho-GSK3β staining in chondrocytes had only an extranuclear pattern (Fig 1B). An high level of staining was also found in calcified cartilage areas, where terminally differentiated chondrocytes survive, suggesting that a similar phenotype had been improperly recapitulated in OA articular cartilage (bottom 10x and 40x detail of the IHC results shown in the lower panels of Fig 1A).

### GSK3 inactivation *in vitro* determines ROS production and oxidative damage

**Fig 2:**
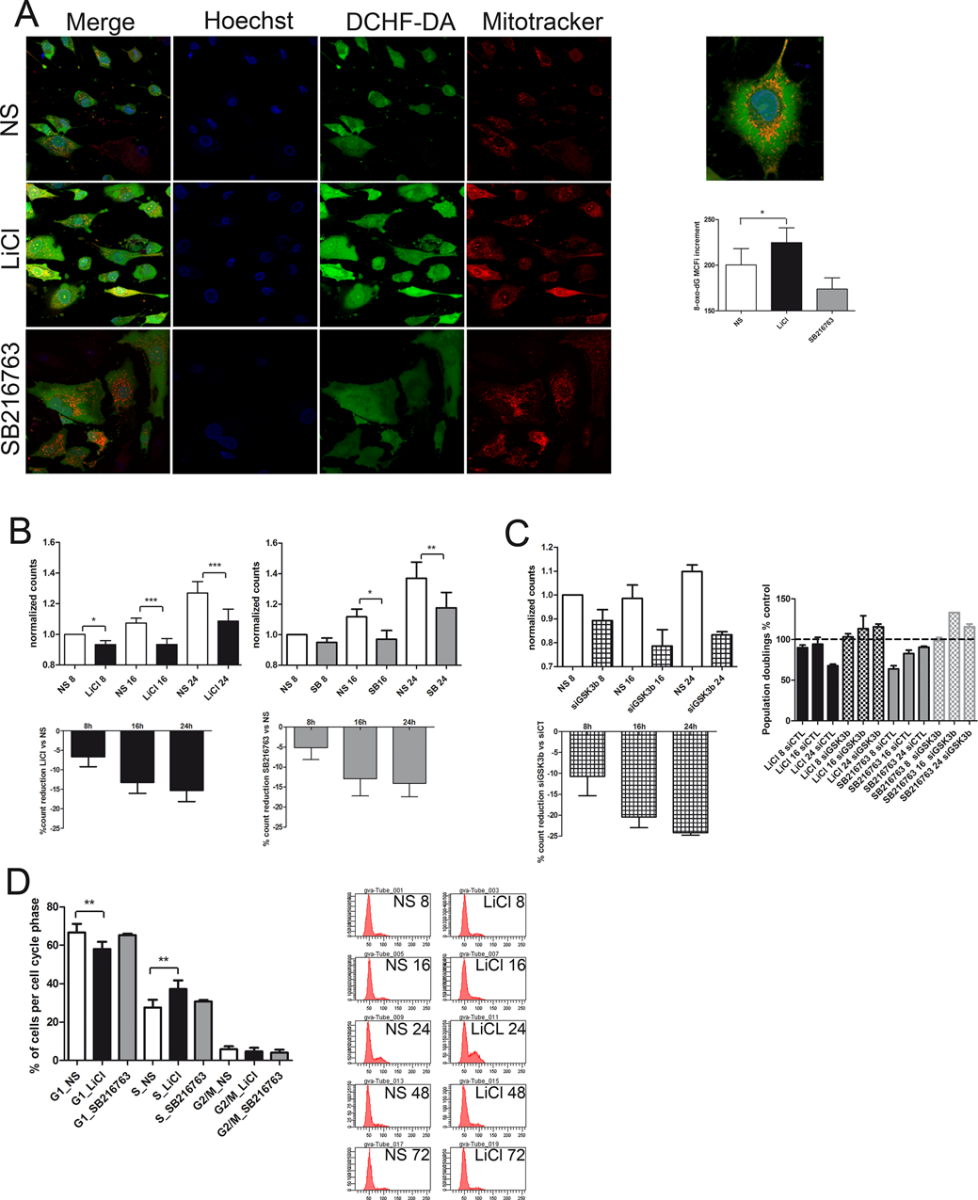
*In vitro* pharmacological inhibition of GSK3β determines ROS production, oxidative damage, stress dependent growth inhibition and activation of an intra-S checkpoint. **2A**, Compared to control (NS), 5 mM LiCl and 10 μM SB216763 increases ROS production and mitochondria activation at 4 hours treatment. Merged and separated signals of Hoechst 33258 nuclear counterstaining, DCHF-DA and Mitotracker Orange CMTMRos mitochondrial staining. Right: high magnification detail of a LiCl treated cell. Right Graph: at 16 hours, LiCl (black columns) but not SB216763 (grey columns) induced a significant increased (n = 5) accumulation of 8-oxo-dG compared to controls (white columns) on the basis of a flow cytometry analysis. **2B**, LiCl (black columns) and SB216763 (grey columns): longitudinal assessment of the effects of GSK3β inhibition on cell growth versus the control (white columns). Upper graphs: counts normalized versus the 8 hours count; lower graph: percentage count reduction due to either LiCl or SB216763 at each time point. **2C**, longitudinal assessment of the effects of siRNA mediated GSK3β silencing (squared columns) on cell growth versus the control non targeting siRNA (white columns). Right graph: Population doublings reduction following either 5 mM LiCl or 10 μM SB216763 stimulation as percentage of each unstimulated control at 8, 16 and 24 hours of both siCTL and siGSK3β treated cells **2D**, Left: LiCl determines a significant increased () percentage of cells in the S phase at 24 hours, as evidenced by DNA staining (Sytox green, n = 9 different experiments with LiCl and 4 with SB216763). Right: a representative example with cell cycle analysis of control (left) versus 5mM LiCl (right) treated cells at each time point. *P < 0.05; **P < 0.01;***P < 0.001.

In monolayer cultures at log phase, GSK3β inactivation with either LiCl or SB216763 determined increased ROS production in activated mitochondria as detected by combining the green DCHF-DA ROS probe with the red Mitotracker Orange CMTMRos mitochondrial probe, that yielded an orange staining (Fig 2A). Noteworthy, besides the increased DCHF-DA signal, the increased Mitotracker CMTMRos signal is a confirmation that the treatment with both the GSK3β inhibitors induces ROS production, since Mitotracker signal is increased by these species [16]. Confocal microscopy analysis revealed interesting morphological features: in most chondrocytes the overlapped staining had a perinuclear pattern, ROS also accumulated in the nucleus (see high magnification image in Fig 2A) and some characteristic nuclear mitotracker stained spots became evident in treated cells (right images in Fig 2A).

To assess the effect of increased ROS production, chondrocytes were stained for 8-oxo-dG, a well known oxidative damage marker [23]. At 16 hours, the LiCl treated cells accumulated a significantly higher level of 8-oxo-dG compared to control cells (p = 0.024, n = 5; S2 File).

### GSK3 inactivation affects cell proliferation with S-phase arrest

LiCl dependent GSK3β inactivation impacted on cellular proliferation, with significant cell count reduction at 8, 16, 24. Fig 2B left graph (and S2 File) shows the cumulative normalized results of experiments performed with cells from different patients. Similar results were obtained using SB216763 (Fig 2B right graph and S2 File), with significantly reduced counts at 16 and 24 hours. For both GSK3β inhibitors, the percentage reduction was maximal at 24 hours, approaching 15% (Fig 2B, lower graphs).

To confirm that GSK3β activity impacts on cell proliferation experiments were also carried out with GSK3β siRNA. Silencing efficiency was very high at both mRNA (83% as assessed by real time PCR analysis of GSK3β expression in siGSK3β versus siCTL chondrocytes) or protein level (89% as assessed by Flow cytometry detection of GSK3β). GSK3β silencing reproduced the effect of pharmacologic GSK3β inhibition at 8, 16 and 24 hours with regards to reduction in cell proliferation (Fig 2C and S2 File) and in the presence of GSK3β silencing, the effects of LiCl or SB216763 are abolished with regards to proliferation reduction. Fig 2C right graph (and S2 File) shows the Population doublings reduction following either 5 mM LiCl or 10 μM SB216763 stimulation as percentage of each unstimulated control at 8, 16 and 24 hours of both siCTL and siGSK3β treated cells [24]. Sytox green DNA staining confirmed a significant cell accumulation in S phase at 24 hours, following treatment with LiCl. Fig 2D shows the cumulative (n = 9 different experiments with LiCl and 4 with SB216763, see also S2 File) distribution of the cells in each cell cycle phase, and a representative case with data for each time point, on the right.

### GSK3 inactivation is responsible for chondrocyte senescence and hypertrophy

**Fig 3:**
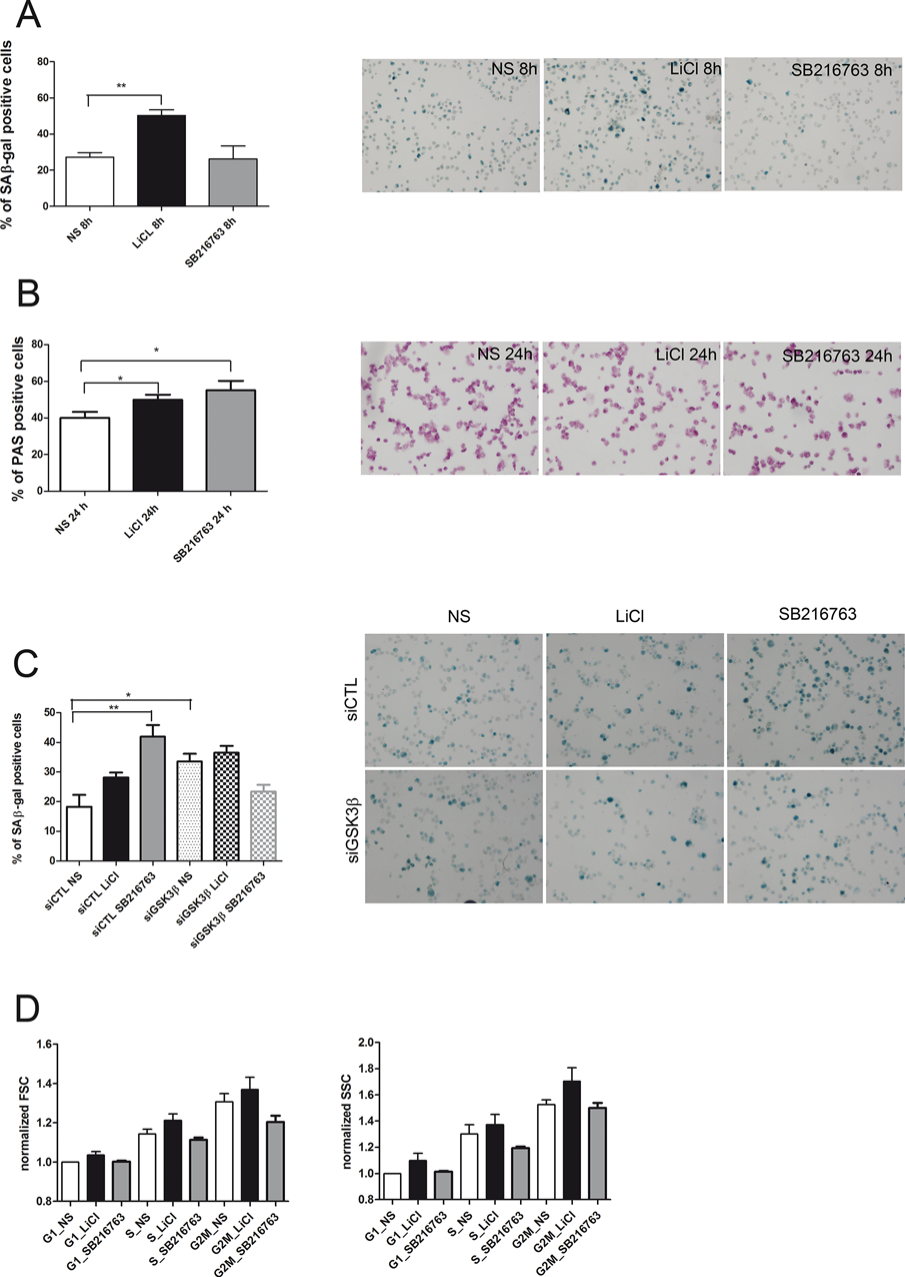
Effects of GSK3β inhibition on senescence markers and scatter properties. SA-β Galactosidase activity and PAS staining were quantitatively evaluated by image analysis: cells were automatically detected by SyBr Green nuclear counterstaining and the percentage of cells above a given staining intensity threshold was determined. **3A**, SA-β Galactosidase activity. 5mM LiCl increases the percentage of SA-β Gal positive cells already at 8 hours of treatment (n = 6 different experiments with LiCl and 4 with SB216763). Right images: representative pictures of non stimulated (NS) and LiCl or SB216763 treated cells (10x original magnification). Hypertrophic cells also show the strongest level of SA-β Gal activity. **3B**, PAS staining. A significant increase in PAS staining was observed in chondrocytes treated with 5mM LiCl (n = 5) or 10μM SB216763 (n = 4) at 24 hours. Right image: representative pictures showing that hypertrophic cells also has the strongest level of PAS staining (10x original magnification). **3C**, SA-β Galactosidase activity is increased by GSK3β silencing after 24 hours. **3D**, Cell cycle phase distribution of Forward Scatter (left graph, a parameter related to cell size) and Side scatter (right graph, a parameter related to cell granularity) of different experiments (n = 8 with LiCl and 4 with SB216763), with values normalized to that of each control G1 phase cells. 5mM LiCl treatment determines an increase of scatter values at G1, S and G2M phase. *P < 0.05; **P < 0.01;***P < 0.001.

GSK3β inactivation induced chondrocyte senescence as assessed by the significant increase of SA-β Gal activity [17] after 5mM LiCl. Noteworthy, the cells with stronger SA-β Gal staining were larger and with a “hypertrophic” phenotype. A quantitative analysis of the increased percentage of senescent/hypertrophic cells was undertaken at 8, 16 and 24 hours and indicated a significant increase already at 8 hours (Fig 3A and S3 File). GSK3β inactivation by either LiCl or SB216763 also led to glycogen accumulation: the number of PAS positive cells was significantly higher at 24 hours for both 5mM LiCl and 10μM SB216763 (Fig 3B and S3 File). Compared to SB216763, 5mM LiCl was a more effective stimulus for glycogenesis, since treated cells had a significantly higher PAS staining already at 16 hours. Interestingly, at 24 hours LiCl treatment, hypertrophic chondrocytes showed both stronger level of SA-β Gal and of PAS staining. A flow cytometric analysis combining cell cycle information and light scattering properties confirmed that already at 8 hours stimulation at each cell cycle phase, LiCl treatment led to the accumulation of chondrocytes larger than control and richer of intracellular structures that can reflect the light, as evidenced by their increased forward and side scatter [25], respectively (Fig 3C and S3 File). We also obtained a statistically significant higher level of SA-β Gal staining in siGSK3β compared to siCTL chondrocytes, while in siGSK3β cells, the addition of either LiCl or SB216763 did not increase further the level of senescence (Fig 3C and S3 File).

### GSK3 inactivation results in a DNA damage response and LiCl increases IKKα expression

**Fig 4:**
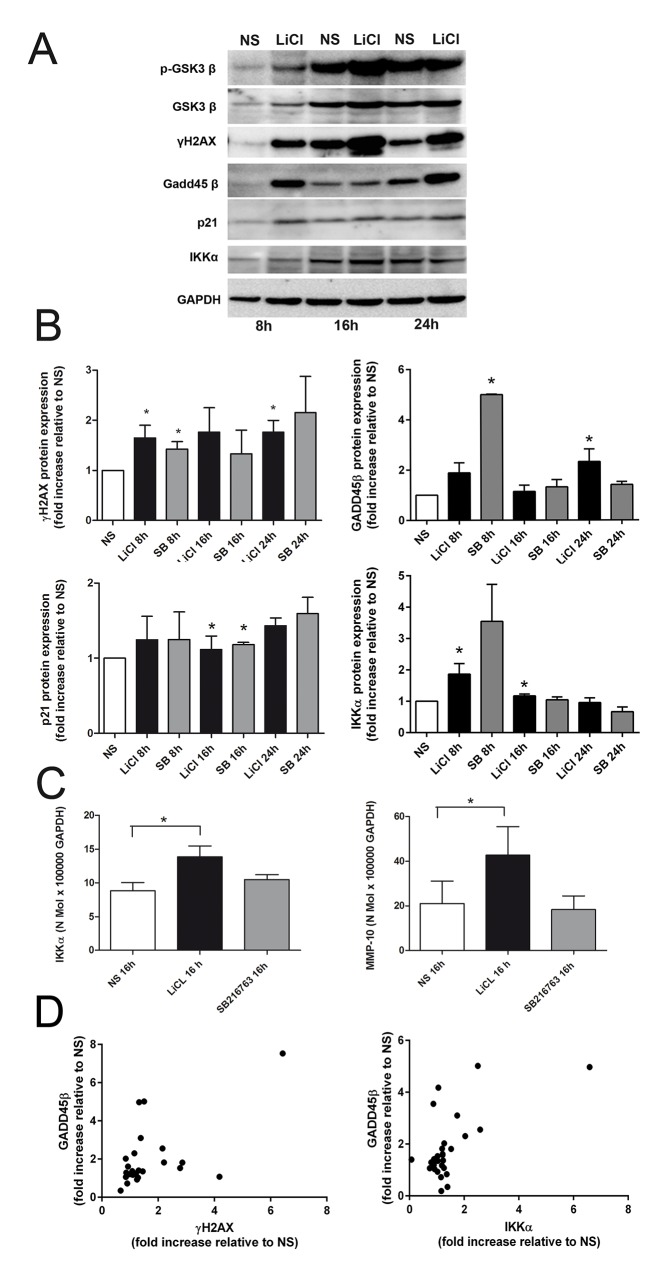
LiCl treatment determines a DNA damage response and increases expression of markers of chondrocyte differentiation. **4A**, LiCl mediated increased GSK3β phosphorylation leads to a DNA damage response (DDR) (a representative example of one out of several experiments). The DDR includes markers of DNA damage (double strand breaks evidenced as γH2AX), increased expression of GADD45β and p21. **4B**, Cumulative densitometric analysis following either 5mM LiCl (LiCl) or 10μM SB216763 (SB) treatment (“n” of experiments detailed within brackets for LiCl and SB216763) of the “fold change increase” in comparison of not stimulated samples (NS) of γH2AX (7 patients for LiCl and 5 for SB216763), GADD45β (6,2), p21 (7,3). IKKα (8,4) was also evaluated. **4C**, 16 hours LiCl treatment significantly increased gene expression of IKKα (n = 3) and of its target gene MMP-10 (n = 3). **4D**, Cumulative correlation analysis of the fold changes protein expression values, considering all the samples independently of time and stimulus, indicated the strong association between the hypertrophy marker GADD45β and both γH2AX (left graph: Spearman r value = 0.48, p = 0.0066, n = 25) and IKKα (right graph: Spearman r value = 0.37, p = 0.024, n = 30). *P < 0.05; **P < 0.01;***P < 0.001.

As expected, LiCl or SB216763 were effective in increasing the extent of phosphorylated GSK3β. Noteworthy, the treatment also induced a slight increase of total GSK3β expression, that also appeared to change in non stimulated cells at 16 and 24 hours, reflecting cell cycle progression. We then performed western blot analysis to investigate whether the LiCl induced increased S phase could be dependent on an activated intra S checkpoint following DNA damage [26]. As shown in the representative case in Fig 4A, LiCl leads to a DNA damage response (DDR), with increased expression of γH2AX, GADD45β and p21. Fig 4B and S4 File show the cumulative densitometric analysis of the level of these proteins following either 5mM LiCl or 10μM SB216763 treatment. γH2AX level was increased at 8 hours by both LiCl and SB216763 and at 24 hours by LiCl. Moreover, γH2AX increase was an effective stimulus for the early induction of GADD45β, significantly increased by SB216763 at 8 hours and by LiCl at 24 hours. GADD45β dependence on γH2AX was further confirmed by the strong correlation between the two proteins (Fig 4D, left graph and S4 File). GADD45β, in turns, led to an increased expression of p21, a marker of senescence [27], significantly induced at 16 hours by both LiCl and SB216763. The effects of GSK3β inactivation on cell growth prompted us to investigate regulation of IKKα, involved in chondrocyte proliferation [28]. Fig 4B shows the cumulative results of 8 (LiCl) or 4 (SB216763) independent experiments and indicates that LiCl induces a modest, yet significant increase of IKKα protein expression at both 8 and 16 hours. In keeping with these findings, at 16 hours stimulation, 5mM LiCl but not 10 μM SB216763 significantly increases IKKα mRNA expression (Fig 4C and S4 File), and of its target gene MMP-10 (Fig 4C and S4 File). IKKα was also found to correlate with GADD45β expression (Fig 4D, right graph and S4 File).

### Evidence of a DNA damage response associated with GSK3 inactivation in cartilage

In mid-deep layers of cartilage samples derived from obese OA patients, we found occurrence and stronger staining of the axis “oxidative DNA damage>GADD45β>p21”, responsible for both senescence and hypertrophy after GSK3β inactivation in vitro.

**Fig 5:**
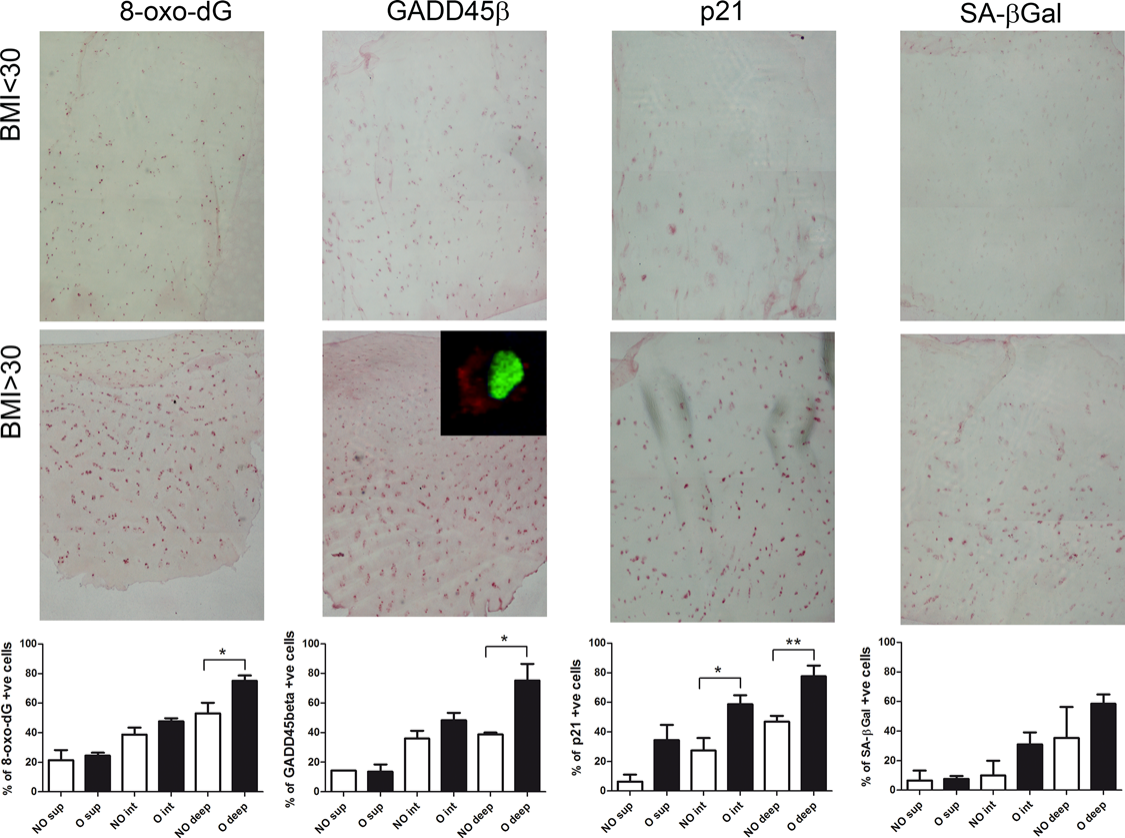
Association of oxidative damage, markers of DDR and senescence in mid-deep layers of cartilage of obese patients. 8-oxo-dG, GADD45β, p21 and SA-β-Gal assessed with immunohistochemistry and colorimetric detection and bright field images at 4x original magnification: cases representative of non obese (upper panel) or obese (lower panel) patients. High magnification inset shows the prevalent cytoplasmic localization of GADD45β signal (red staining) with sybr green as a nuclear counterstaining. For each marker, experiments were carried out in the same experimental session to rule out biases in comparing the signal intensity. Below the pictures for each marker a cumulative assessment (with mean and standard error of mean) of the percentage of positive cells in superficial, intermediate and deep layers in non obese (NO, white column) or obese patients (O, black columns) is shown. *P < 0.05; **P < 0.01;***P < 0.001.

Oxidative DNA damage was investigated by mean of 8-oxo-dG staining [23]. Overall, in cartilage of obese patients we found association of higher staining of phospho-GSK3β (as detailed in Fig 1), 8-oxo-dG, GADD45β (with an exclusive cytoplasmic distribution, see inset in Fig 5) and of its target gene p21, and of immunologically detectable SA-β Gal (See Fig 5, lower right panel), suggesting that also in the tissue there is evidence of a mechanistic link between GSK3β inactivation, DNA damage response and chondrocyte senescence. Fig 5 shows correlated immunohistochemistry experiments on cartilage sections derived from two representative OA cartilage samples resulted negative (upper panel) or positive (lower panel,) for phospho-GSK3β. Bottom graphs show the image analysis of the percentage of positive cells for the different markers in the different superficial, intermediate and deep cartilage zones in samples derived from non obese and obese patients (S5 File). Obese patients presented significantly higher level of 8-oxo-dG, GADD45β and p21 in deep cartilage layers.

### Insulin mimicks lithium effects on chondrocyte cultures

Given the significant association between the BMI and the percentage of phospho-GSK3β chondrocytes, we investigated on the factors that can be responsible for the GSK3β inactivation in vivo. Insulin is a likely candidate, known to be frequently increased in obese patients because of insulin resistance. Insulin shares with LiCl the ability to inactivate GSK3β through the PI3K/Akt pathway [29, 30] although LiCl is able to inactivate GSK3β through multiple mechanisms [30]. Therefore, we studied the effects of 33 nM insulin [18] on chondrocyte proliferation. Similarly to 5mM LiCl, insulin led to decreased chondrocyte proliferation at 8, 16 and 24 hours (S3 File).

## Discussion

The hypothesis of our work is that GSK3β inactivation can sustain chronic impairment of articular chondrocytes, via a mitochondrial mechanisms [31] that leads to ROS production and cellular senescence.

DNA damage has been found in osteoarthritic samples, in conjunction with a progressive/stress-induced senescence [32]. This condition is worsened by the association of a metabolic syndrome, since obesity is accompanied by a systemic DNA damage response which sustains chronic inflammation [33, 34] and degeneration of multiple post-mitotic tissues. We indeed found the extent of GSK3β inactivation in OA cartilage correlated with BMI, recently recognized together with age as one of the potential predictor factors for knee osteoarthritis [10].

One of the critical criteria for metabolic syndrome is insulin resistance [8]. In this case fat and muscle cells are unable to respond to insulin that increases as a compensatory mechanism and mediates adverse effects in different tissues including cartilage. With regards to signal transduction, it is worth noticing that insulin is able to activate the PI3K/-Akt pathway which then inactivates GSK3β.

To tease out the consequences of GSK3β inactivation in chondrocytes, we performed several in vitro experiments with both the pharmacological inhibitor LiCl and the specific inhibitor SB216763. The latter indeed had been tested against a panel of 25 different serine/threonine and tyrosine protein kinases that showed little or no inhibition [15].

The two substances were used at concentrations close to those found to exert similar effects on glucose incorporation, stimulation of glycogen synthase activity and transcription of β-catenin-LEF/TCF regulated reporter gene [15].

Since LiCl is able to activate the PI3K/-Akt pathway and to inhibit GSK3β [30, 35], it can be considered as a mimicker of insulin or other growth factors or inflammatory cytokines that lead to GSK3β inactivation as a result of PKC/PI3K/Akt activation [36, 37], whereas the comparison with SB216763 and GSK3β silencing strategies can help in distinguishing the effects which are uniquely dependent on GSK3β inactivation versus those which are instead dependent on other signaling molecules.

We used log phase chondrocyte cultures to investigate the effects of GSK3β inactivation on chondrocyte proliferation, reported to occur in osteoarthritic cartilage as an attempt to keep tissue homeostasis [38]. We found that GSK3β inactivation is responsible for chondrocyte senescence as detected by impaired proliferation or accumulation of SA-β Gal [39]. We then moved to investigate the molecular mechanisms and found that LiCl or SB216763 dependent GSK3β inactivation in mitochondria is responsible for sustained ROS production as previously described [31]. Interestingly, we observed occurrence of mitochondrial-nuclear translocation of proteins selectively stained by Mitotracker. It has been reported that during the stress response heat shock proteins functioning as chaperones migrate to the nucleus bound to anti-apoptotic proteins [40]. Therefore, GSK3β inhibition activates compensatory activities of the cells to protect themselves from oxidative stress.

Intracellular ROS generation then damages DNA and triggers a DNA damage response, known to be involved in senescence induction and maintenance [26].

GSK3 inhibition indeed increased γH2AX, the marker of double strand breaks, corresponding to the phosphorylated form of the histone H2AX, that tags double strand breaks in DNA, to organize the multimeric protein complex for DNA repair. The DNA damage determines induction of GADD45β, a growth arrest and DNA damage inducible gene that plays an important role in chondrocyte terminal differentiation [41] and reported to drive chondrocyte hypertrophy and prevent apoptosis of hypertrophic chondrocytes [42]. GADD45β could participate in cell cycle arrest being responsible for both an intra S checkpoint [43], such as that observed in our LiCl treated cultures and induction of p21 [27], the cyclin dependent kinase inhibitor (CKI) induced following DNA damage [26] and involved in cell cycle arrest and DNA repair.

DNA damage was also assessed considering 8-oxo-dG staining, one of the best characterized marker of nuclear and mitochondrial oxidative stress [23]. Noteworthy, mitochondrial DNA is much more susceptible than nuclear DNA to oxidative damage because of enhanced ROS formation and reduced repair capacity [44]. We easily detected increased γH2AX following GSK3β inhibition, but 8-oxo-dG increased only after LiCl and not SB216763. SA-β Gal [17] and PAS staining [19] were then used not alone, but in combination to tease out the differential effects downstream GSK3β inhibition. Investigation of both aspects allowed us to distinguish the effects on either true senescence or hypertrophy. Noteworthy, only LiCl and not SB216763 led to increased cell expression of SA-β Gal. However, GSK3β inactivation by means of silencing strategies was effective in producing a significant increase of cell senescence, possibly because of the greater magnitude of the effect compared to SB216763. At the same time cell scatter properties indicated increased cell size and granularity in cells treated with LiCl. On the other hand, as expected due to the effect on glycogen synthase [15], both LiCl and SB216763 increased the percentage of PAS positive cells. Enhanced glycogenesis has been linked to cellular senescence and aging [19] and also proposed as a marker of hypertrophic chondrocytes [45]. Overall, the above findings suggest that induction of senescence may require a critical level of ROS generation that in our experimental setting has only been achieved by LiCl. Indeed, LiCl is able to induce ROS not only because of GSK3β inhibition [31] but also because of additional signaling mechanisms such as the activation of PI3K/Akt [30, 46], as downstream the action of insulin [29] or growth factors that drive chondrocyte differentiation It is worthy to underline that the PI3K/Akt pathway has been recently connected with the activation of the NADPH oxidase family of enzymes [46].

Therefore, following LiCl treatment, senescence and hypertrophy overlap in chondrocytes. In keeping with this concept, a link between senescence and articular chondrocyte terminal differentiation has already been reported [47]. Indeed, chondrocyte stimulation with LiCl is known to be associated with accumulation and transcriptional activity of β-catenin which causes progression from the healthy articular toward a more terminally differentiated phenotype [48].

Recently, LiCl has been suggested to protect against cartilage degradation in osteoarthritis [14, 49]. Our findings instead show that LiCl could have deleterious effects on cartilage homeostasis. Indeed, despite its inhibiting activity on NF-κB [14] or p38 MAPK [49] pathways, LiCl promotes not only chondrocyte hypertrophy but also senescence and increased gene/protein expression of IKKα and increased gene expression of its target MMP-10, pivotal in ECM remodelling and chondrocyte terminal differentiation in both human and murine chondrocytes.

A similar association of phospho-GSK3β>oxidative stress>GADD45β>p21 was found in cartilage of obese patients whose mid-deep layers presented markedly increased levels of 8-oxo-dG rather than γH2AX that represents a transient phenomenon [50, 51].

Chondrocyte susceptibility to oxidative damage is well known as well as implication for ROS in intrinsic senescence of OA tissues. Oxidative stress is in turn the major determinant underlying the “extrinsic” or stress-induced senescence of aging chondrocytes [52, 53] and according to the findings of the present paper, of chondrocytes in cartilage of obese OA patients.

We recently reported that progressed differentiation, increased extracellular matrix remodeling and senescence can coincide in chondrocytes exposed to chronic inflammatory stimuli which are responsible for enhanced expression of p16, one of the two CKI involved in senescence dependent cell cycle arrest [47]. In this paper we instead show that following GSK3β inactivation, increased intracellular ROS trigger a DNA damage response which leads to an up-regulated expression of p21, the other CKI. It is noteworthy that the “postmitotic” status of healthy articular chondrocytes features an “housekeeping” expression level of the two CKIs, which may change in OA. p16 is increased in OA chondrocytes compared to aged-matched cells [54]. With regards to p21, conflicting data have been reported in literature. p21 has been found to increase as a function of senescence in SAMP mice as a result of increased GADD45β expression [27]. The up-regulated p21 expression has been mechanistically correlated to the increased caveolin expression found in both human and rat OA cartilage [55]. Conversely, Rose found decreased p21 in late OA samples, possibly because these were severely degenerated OA tissues [32]. In the present paper we collected several evidences that in vitro LiCl mediated GSK3 inactivation leads to chondrocyte hypertrophy in conjunction with cell senescence as indicated by increased p21 expression. Our findings are in keeping with a previous report which pointed at an association between increased p21 expression and increased chondrocyte hypertrophy in both growth plate and articular cartilage [56]. We also found a high p21 expression in cartilage of obese patients that also present high cell senescence as shown by SA-β gal staining.

## Conclusions

In summary, we provide several evidences that GSK3β activity can be compromised *in vivo* in cartilage of obese OA patients and our *in vitro* experiments indicate that it is instead required for chondrocyte mitochondrial function, tissue homeostasis and prevention of oxidative damage, hypertrophy and senescence. Therefore our findings question the use of LiCl in OA treatment.

## Supporting Information

S1 File Data used in Fig 1. (XLSX)

S2 File Data used in Fig 2. (XLSX)

S3 File Data used in Fig 3. (XLSX)

S4 File Data used in Fig 4. (XLSX)

S5 File Data used in Fig 5. (XLSX)
